# Association of the *KCNJ11* E23K (rs5219) variant with proliferative diabetic retinopathy in Lebanese patients with type 2 diabetes

**DOI:** 10.3389/fmed.2026.1874270

**Published:** 2026-07-08

**Authors:** Rita Nemr, Akram Echtay, Perizat Kanabekova, Zhansaya Bauyrzhanova, Dana Amanzhol, Wassim Y. Almawi

**Affiliations:** 1 Department of Internal Medicine, LAU Medical Center - Rizk Hospital, Beirut, Lebanon; 2 Department of Adult Endocrinology and Diabetes, Rafic Hariri University Hospital, Beirut, Lebanon; 3 Department of Biomedical Sciences, Nazarbayev University School of Medicine, Astana, Kazakhstan; 4 Faculty of General Medicine, Astana Medical University, Astana, Kazakhstan; 5 Faculty of Sciences, El-Manar University, Tunis, Tunisia

**Keywords:** diabetic retinopathy, *KCNJ11* gene, Kir6.2, potassium channel, type 2 diabetes

## Abstract

**Background:**

ATP-sensitive potassium channels regulate insulin release, with the *KCNJ11* gene encoding the Kir6.2 channel’s pore subunit. The E23K variant, previously linked to type 2 diabetes (T2DM), was examined in a large Lebanese cohort to determine its relationship to diabetic retinopathy (DR) severity and glycemic control.

**Methods:**

A case–control sample of 1,415 adults with T2DM were classified as diabetes without retinopathy (DWR; *n* = 933), non-proliferative DR (NPDR; *n* = 342), or proliferative DR (PDR; *n* = 140) based on ETDRS criteria, and compared with 1,389 normoglycemic controls. Logistic regression was used to assess genetic associations across multiple inheritance models. Sensitivity analyses stratified by diabetes duration and area under the ROC curve (AUC) compared discrimination using age and sex, with or without genotype.

**Results:**

The K allele was more frequent in T2DM than in controls. Although E23K was not associated with overall DR, its effect was stage-specific. In comparisons of PDR with DWR, each additional K allele increased PDR odds, and K/K homozygosity showed a stronger recessive effect. The K/K genotype was notably enriched among DR patients with HbA1c ≤ 7.0%, indicating an elevated risk under good glycemic control, but showed no signal in those with poorer control. It was also overrepresented in PDR. Risk associations were more pronounced in individuals with diabetes duration ≥10 years, and adding genotype information resulted in a small increase in model discrimination beyond age and sex alone (AUC = 0.552–0.598; ΔAUC = 0.046). However, the overall discriminatory performance remained poor, suggesting limited clinical utility of E23K genotyping as an isolated predictive marker.

**Conclusion:**

In this Middle Eastern population, *KCNJ11* E23K showed a stage-specific association with prevalent proliferative, vision-threatening DR rather than with earlier stages of retinopathy. This supports the potential utility of E23K as a marker of advanced retinopathy severity, although longitudinal studies in diverse ethnic groups are required to determine its relationship with disease progression.

## 1 Introduction

Diabetic retinopathy (DR) is a major microvascular complication of diabetes and a leading cause of visual impairment among working-age adults worldwide (1–3). Its clinical spectrum ranges from non-proliferative DR (NPDR) to proliferative DR (PDR), the latter defined by pathological retinal neovascularization and responsible for most vision-threatening outcomes (4, 5). Diabetic macular edema may occur at any stage and reflects vascular leakage and ischemia (6, 7). Although chronic hyperglycemia is the dominant modifiable driver, hypertension, dyslipidemia, body mass index (BMI), and diabetes duration also contribute to disease onset and progression (6, 8). Nevertheless, considerable inter-individual variation persists even after accounting for metabolic factors, underscoring the heritable component of microvascular susceptibility (9–12). Genetic factors, including single-nucleotide polymorphisms (SNPs), were reported to exert a greater influence on the development of PDR than on the earliest retinal changes, suggesting that they are more likely to determine the tempo of disease progression rather than to trigger the initial abnormalities.

Growing interest has shifted toward genes that regulate β-cell physiology, as disturbances in insulin secretion and metabolic load are closely linked to the progression of retinopathy. *KCNJ11* encodes Kir6.2, the pore-forming component of the ATP-sensitive potassium channel (K_ATP_) in pancreatic β-cells, a channel that links intracellular ATP/ADP balance to membrane depolarization, calcium entry, and controlled insulin release (13–15). Variants that shift the channel’s ATP sensitivity or gating behavior can weaken glucose-driven insulin release and place β-cells under persistent metabolic pressure, a state that promotes oxidative damage, endothelial dysfunction, and inflammatory changes implicated in DR (16, 17). Given that β-cell strain and swings in glycemia are major contributors to microvascular injury, genes governing K_ATP_ channel activity are well-positioned to influence the severity of retinopathy.

The E23K (rs5219) variant is a missense mutation in *KCNJ11* exon 1, resulting from C → T substitution and corresponding lysine → glutamate substitution at position 23 in the Kir6.2 N-terminal (cytosolic) region. This contributes to ATP-dependent channel closure, and the lysine substitution reduces ATP sensitivity, thereby favoring channel opening under normal metabolic conditions (17). This shift reportedly enhances K_ATP_ channel activity, weakens glucose-evoked insulin secretion, and increases metabolic strain in allele-carrying individuals (15, 18). E23K is also one of the most consistently reproduced type 2 diabetes (T2DM) risk variants across diverse populations, including Arab populations (19–24). Beyond diabetes risk, E23K has been implicated in accelerated β-cell dysfunction, glycemic instability, and downstream complications, although evidence for microvascular outcomes remains inconsistent, with studies reporting weak or null associations, often limited by small sample sizes, heterogeneous phenotyping, or lack of severity stratification (23, 25–28).

Given the high regional burden of diabetes and the limited genetic epidemiology data from the Middle East, clarifying the relationship between E23K and DR severity is clinically important. Variants that affect β-cell stress and metabolic load may show stronger associations with advanced retinopathy stages characterized by ischemia-driven pathology (29). Using standardized Early Treatment Diabetic Retinopathy Study (ETDRS) grading in a large Lebanese cohort (30), we examined the association of E23K with overall DR, stage-specific effects across NPDR and PDR, and interactions with metabolic exposure. We hypothesized that E23K would demonstrate stronger associations with prevalent proliferative diabetic retinopathy than with earlier stages of retinopathy.

## 2 Subjects and methods

### 2.1 Study subjects

The case–control study enrolled 2,804 participants, including 1,389 normoglycemic controls and 1,415 individuals with T2DM. Among those with diabetes, 482 had DR and 933 had no retinal involvement (DWR). Type 2 diabetes was diagnosed on clinical grounds; no participant presented with ketoacidosis at onset, and all insulin-treated individuals had been managed with oral agents for at least 2 years. Participants were excluded if they had type 1 diabetes or latent autoimmune diabetes in adults (LADA), non-diabetic causes of retinopathy (such as retinal vein occlusion, age-related macular degeneration, or hypertensive retinopathy), significant media opacities limiting adequate fundus visualization, severe renal impairment (estimated glomerular filtration rate [eGFR] < 30 mL/min/1.73 m^2^), or incomplete clinical or genetic data. Cases with missing values for variables required in multivariable analyses were excluded using a complete-case approach. Detailed information on antidiabetic treatment regimens, including insulin, oral hypoglycemic agents, and combination therapies, was not consistently available for all participants and was therefore excluded from the analysis.

### 2.2 Assessment of diabetic retinopathy

Clinical assessment of DR was conducted by trained ophthalmologists who were blinded to laboratory data. Assessment of DR was performed under dilated conditions with indirect ophthalmoscopy and slit-lamp biomicroscopy employing a 90-diopter lens. The retinal evaluation focused on characteristic microvascular lesions, including microaneurysms, intraretinal hemorrhages, hard exudates, cotton-wool spots, intraretinal microvascular abnormalities, and retinal or optic-disc neovascularization. DR severity was graded according to the ETDRS criteria (30), with independent assessments and consensus review to resolve any discrepancies.

### 2.3 Biochemical analysis

Venous blood was drawn after an overnight fast and distributed into additive-specific vacutainer tubes according to the required analyses. Glucose was measured in sodium fluoride–potassium oxalate tubes using the hexokinase method on the Roche Cobas integra 800 (Roche, Mannheim, Germany). Total hemoglobin and HbA1c were quantified in EDTA-anticoagulated blood on cobas® c311 (Roche). Serum obtained from separator tubes was used to measure total cholesterol, LDL-cholesterol, HDL-cholesterol, and triglycerides on cobas® c502 linked to cobas® c6000 (Roche). Creatinine was measured by the Jaffe reaction, and additional liver and renal function tests, as well as electrolyte tests, were performed on Dade-Behring platforms.

### 2.4 E23K (rs5219) genotyping

Genomic DNA for *KCNJ11*/Kir6.2 (rs5219) genotyping was isolated from peripheral venous blood samples of cases and controls using the QIAamp DNA blood Mini kit (Qiagen, Hilden, Germany). Allelic discrimination was performed on the applied biosystems StepOne plus real-time PCR system using VIC/FAM-labeled TaqMan primers and probes, following the manufacturer’s protocols (applied biosystems; ThermoFisher scientific, Waltham, MA, USA); genotyping efficiency was 100%. Blinded replicate samples were included as quality controls and showed >99% concordance. The homozygous major-allele genotype (E/E) was used as the reference category (odds ratios [ORs] = 1.00) to estimate ORs and 95% confidence intervals (CIs) for overall DR risk. The observed genotype frequencies for the E23K polymorphism were consistent with the hardy–Weinberg equilibrium (HWE).

### 2.5 Statistical analysis

Power calculations assuming a 30% minor allele frequency, an OR of 1.5, 80% power, and a significance level of 0.05 indicated that a minimum of 400 DR cases and 800 controls would be required. Our dataset, which included 482 DR cases and 933 DWR controls, exceeded these thresholds. The PDR subgroup (approximately 150–200 individuals) also retained sufficient power to detect moderate-to-large genetic effects (OR ≥ 1.8). Allele frequencies were determined by direct genotype counting, and all groups conformed to HWE expectations (*p* > 0.05) as assessed by a *χ*^2^ goodness-of-fit test. Differences in allele and genotype frequencies between T2DM cases and controls were evaluated with Pearson’s *χ*^2^ test. Continuous variables were summarized as mean ± SD when normally distributed using the Shapiro–Wilk test and compared using Student’s *t*-test. Appropriate non-parametric tests were used for variables that deviated from normality; homogeneity of variances was evaluated using Levene’s test. The relatively large standard deviations observed for selected variables reflect underlying clinical heterogeneity rather than data irregularities. Missing data were assessed prior to analysis, and participants with incomplete genotyping or missing values for variables included in regression models were excluded from the corresponding analyses, using a complete-case approach. Given the low proportion of missing data and the cross-sectional study design, no multiple imputation procedures were performed.

Associations between E23K genotypes and clinical outcomes were assessed using logistic regression. Multinomial models compared genotype categories, while binary logistic models contrasted DR with DWR. Analyses of T2DM cases and controls were adjusted for BMI, blood pressure, hypertension status, and lipid measures, whereas comparisons between the DR and DWR groups were adjusted for age at onset, diabetes duration, family history, hypertension, and lipid profile. Spearman correlation tested relationships between genotypes and clinical traits, and genotype-HbA1c interaction terms were evaluated to identify potential effect modification. Stratified analyses were performed according to glycemic control (HbA1c ≤ 7.0% vs. >7.0%) to assess differences in genetic associations across metabolic states, and stratification by T2DM duration (<10 years vs. ≥10 years) was conducted within regression models to evaluate duration-dependent effects. Benjamini–Hochberg FDR correction was applied to all secondary and exploratory analyses, including HbA1c- and duration-stratified models. Prespecified primary comparisons (DR vs. DWR and PDR vs. DWR) were not adjusted. Secondary results include both nominal and FDR-adjusted *p*-values, with significance defined as an FDR-adjusted *p* < 0.05. Model assumptions, including normality (Shapiro–Wilk test), homogeneity of variances (Levene’s test), multicollinearity (variance inflation factor <5), and, where relevant, proportional odds (Brant test), were verified. Sensitivity analyses using non-parametric methods yielded consistent results (data not shown), supporting the robustness of the findings. All statistical analyses were performed using SPSS 29 (IBM, Armonk, NY). Logistic regression analyses, ROC curve analyses, and Benjamini–Hochberg false discovery rate corrections were conducted within this analytical framework. Two-sided *p*-values <0.05 were considered statistically significant unless otherwise specified.

### 2.6 Ethics and consent

This study was approved by the Institutional Review Board (IRB) of St. Marc Medical Center (protocol number SMMC-2019-0103, dated October 17, 2019) and adhered to the Declaration of Helsinki. Genetic data were pseudonymized by replacing personal identifiers with unique study codes. Informed consent was obtained from all participants to ensure confidentiality and ethical compliance. De-identified data underlying the findings, the full data dictionary, analysis code, and workflow documentation are available at the Mendeley Repository: Doi: 10.17632/c2fjck7ts3.1.

## 3 Results

### 3.1 Study subjects

The study included 1,389 healthy controls, 482 patients with DR, and 933 DWR cases, with comparable geographic and ethnic distribution. As shown in Table 1, mean age and sex did not differ significantly between normoglycemic participants and participants with diabetes, or among diabetic subgroups. Compared with DWR patients, DR cases had an earlier age at diabetes onset, longer disease duration (both *p* < 0.001), and a higher frequency of family history of diabetes (*p* = 0.039). The prevalence of nephropathy was similar between the DR and DWR patient groups. Both systolic and diastolic blood pressure, as well as hypertension prevalence, were significantly higher in T2DM patients than in controls (*p* < 0.001) but did not differ between DR and DWR subgroups. Fasting glucose and HbA1c were elevated in patients with T2DM compared with controls (both *p* < 0.001) but were comparable between DR and DWR. Lipid profiles differed significantly between patients with T2DM and controls, with HDL (*p* = 0.007), LDL (*p* < 0.001), and total cholesterol (*p* = 0.009) also differing between the DR and DWR groups. In contrast, triglyceride levels were comparable between DR and DWR groups (*p* = 0.197; Table 1). The relatively wide dispersion observed for select variables, in particular the duration of diabetes and triglyceride levels, is consistent with the heterogeneous clinical characteristics of the study population. Furthermore, Information on prescribed antidiabetic treatments was incomplete across the cohort and therefore was not incorporated into the present analysis. No additional missing data requiring imputation were identified among the variables included in the final analytical models.

**Table 1:** Demographic and clinical characteristics of patients and controls.^1^

Characteristic	Healthy controls	DR cases	DWR controls	*p* ^2^	*p* ^3^
Gender (M:F)^4^	801 (61.2):588 (38.8)	279 (58.0):203 (42.0)	514 (55.1):419 (44.9)	0.191	0.299
Mean age (years)^5^	59.9 ± 9.1	59.3 ± 11.0	60.9 ± 10.6	0.162	0.104
Mean BMI (kg/m^2^)^5^	23.4 ± 3.4	28.6 ± 4.8	28.0 ± 5.0	<0.001	0.273
Diabetes age of onset (years)^5^	NA	50.6 ± 11.5	56.7 ± 11.5	NA	<0.001
Duration of diabetes (years)^5^	NA	8.5 ± 7.3	6.4 ± 5.5	NA	<0.001
Family history of diabetes^4^	0 (0.0)	238 (49.4)	407 (43.6)	NA	0.039
Nephropathy^4^	NA	192 (39.8)	339 (36.3)	NA	0.198
SBP (mmHg)^5^	121.5 ± 14.1	132.8 ± 13.9	132.0 ± 19.0	<0.001	0.757
DBP (mmHg)^5^	78.0 ± 10.5	81.5 ± 12.6	78.6 ± 15.2	0.026	0.135
Hypertension^4^	419 (30.2)	326 (67.7)	479 (51.3)	<0.001	<0.001
Glucose (mmol/L)^5^	5.2 ± 0.7	11.1 ± 4.3	10.8 ± 4.0	<0.001	0.926
HbA1c (%)^5^	5.1 ± 1.1	9.0 ± 2.4	9.0 ± 2.2	<0.001	0.920
HDL (mmol/L)^5^	1.33 ± 0.39	1.05 ± 0.35	1.14 ± 0.39	<0.001	0.007
LDL (mmol/L)^5^	3.00 ± 1.61	3.66 ± 1.00	3.12 ± 1.33	<0.001	<0.001
Total cholesterol (mmol/L)^5^	4.74 ± 0.99	4.83 ± 1.13	5.11 ± 1.28	0.001	0.009
Triglycerides (mmol/L)^5^	1.46 ± 0.86	2.23 ± 1.47	2.42 ± 1.85	<0.001	0.197

BMI, body mass index; DBP, diastolic blood pressure; DR, diabetic retinopathy; DWR, diabetes without retinopathy; HDL, high-density lipoprotein cholesterol; LDL, low-density lipoprotein cholesterol; NA, not applicable; SBP, systolic blood pressure.

1 Study subjects comprised 1,389 healthy controls, 482 DR cases, and 933 T2DM-with-no DR controls.

2 T2DM and healthy controls were compared using Student’s *t*-test for continuous variables and Pearson’s *χ*^2^ test for categorical variables.

3 DR and DWR groups were compared using Student’s *t*-test for continuous variables and Pearson’s *χ*^2^ test for categorical variables. Non-parametric tests were applied when the normality assumption was violated.

4 Number (percent total).

5 Mean ± SD.

### 3.2 E23K alleles and genotypes distribution

Allele and genotype distributions are presented in Table 2. For clarity, the E23K (rs5219) variant is described using the standard amino acid (E/K) notation. The distribution of E23K alleles and genotypes differed significantly between healthy controls and individuals with T2DM (*p* < 0.001), with the K allele notably more common in both the DR (32.3%) and DWR (31.9%) groups than in controls (24.8%). Genotype distributions followed the same pattern: individuals with T2DM had fewer E/E homozygotes and a higher proportion of E/K and K/K genotypes than controls (*p* < 0.001). In contrast, comparisons between the DR and DWR subgroups showed no meaningful differences in allele frequencies (*p* = 0.320) or genotype patterns, and logistic models found no association between either the E/K (*p* = 0.622) or K/K (*p* = 0.327) genotypes and the presence of retinopathy. Taken together, these results suggest that E23K does not affect the presence of retinopathy when examined without stratification, but its impact becomes apparent once disease severity and glycemic control are considered. However, this finding should be interpreted with caution, as HbA1c likely captures unmeasured clinical and lifestyle influences that are not fully accounted for in the analysis. As such, the results are best regarded as exploratory evidence of heterogeneity rather than as definitive evidence of effect modification.

**Table 2:** Bivariate comparisons and multivariable logistic regression analyses of *KCNJ11* E23K alleles and genotypes in type 2 diabetes and diabetic retinopathy.

	Healthy Controls^1^	DR^1^	DWR^1^	*p* ^2^	Chi square^2^	*p* ^3^	OR (95% CI)^3^
E23K allele							
E	2,089 (75.2)^4^	633 (67.7)	1,260 (68.1)	<0.001	46.990	0.320	1.087 (0.922–1.282)
K	689 (24.8)	331 (32.3)	606 (31.9)				
E23K genotype							
E/E	826 (59.5)	215 (44.6)	436 (46.7)	<0.001	52.011		1.00 (Reference)
E/K	437 (31.5)	203 (42.1)	388 (41.6)			0.622	1.061 (0.838–1.343)
K/K	126 (9.1)	64 (13.3)	109 (11.7)			0.327	1.191 (0.840–1.688)

CI, confidence interval; DR, diabetic retinopathy; DWR, diabetes without retinopathy; HDL, high-density lipoprotein cholesterol; LDL, low-density lipoprotein cholesterol; OR, odds ratio; SBP, systolic blood pressure; DBP, diastolic blood pressure; T2DM, type 2 diabetes mellitus.

1 Study subjects comprised 1,389 healthy controls, 482 DR cases, and 933 T2DM-with-no DR (DWR) controls.

2 Logistic regression analysis comparing participants with T2DM and healthy controls, adjusted for age, sex, BMI, diabetes duration, systolic and diastolic blood pressure, hypertension status, HDL cholesterol, LDL cholesterol, total cholesterol, and triglycerides.

3 Logistic regression analysis comparing DR and DWR groups, adjusted for age at diabetes onset, diabetes duration, family history of diabetes, hypertension status, HDL cholesterol, LDL cholesterol, and total cholesterol. Odds ratios (ORs) and 95% confidence intervals (CIs) are presented relative to the reference category.

4 Number (percent total).

### 3.3 Association of E23K alleles and genotypes with DR severity

Unadjusted bivariate analyses of theE23K variant across DR subtypes revealed a clear stage-specific pattern (Table 3). No significant allelic or genotypic differences were seen between DR and DWR overall, or between NPDR and DWR. Specifically, the allelic comparison (K vs. E) showed no association with overall DR (*p* = 0.332; OR = 1.087 [0.922–1.282]) or NPDR (*p* = 0.149; OR = 0.866 [0.715–1.049]). In addition, genotypic, dominant, and recessive models failed to differentiate NPDR from DWR (all *p* > 0.05). However, a strong association was observed with PDR. In unadjusted analyses, the prevalence of the K allele was significantly higher in PDR compared with DWR (*p* = 1.18 × 10^−5^; OR [CI] = 1.780 [1.383–2.292]). Both heterozygous (E/K vs. E/E: *p* = 1.25 × 10^−3^; OR [CI] = 1.946 [1.293–2.926]) and homozygous variant (K/K vs. E/E: *p* = 7.01 × 10^−5^; OR [CI] = 2.927 [1.748–4.901]) genotypes were significantly associated with PDR. These associations were noted under the dominant (*p* = 6.07 × 10^−5^; OR [CI] = 2.161 [1.470–3.176]) and recessive (*p* = 2.99 × 10^−3^; OR = 2.025 [1.291–3.175]) models. These unadjusted findings suggest that E23K, while not associated with early microvascular changes, is clinically significant only in advanced (proliferative) disease, supporting its use to differentiate proliferative, vision-threatening retinopathy from earlier non-proliferative stages.

**Table 3:** Bivariate analysis of the association between *KCNJ11* E23K alleles/genotypes and diabetic retinopathy severity.

Genetic model	Comparisons	DR vs. DwR^1^		NPDR vs. DwR^1^		PDR vs. DwR^1^	
		*p*	OR (95% CI)^2^	*p*	OR (95% CI)^2^	*p*	OR (95% CI)^2^
Allelic	K vs. E	0.332	1.087 (0.922–1.282)	0.149	0.866 (0.715–1.049)	1.18 × 10^−5^	1.780 (1.383–2.292)
Genotypic	E/K vs. E/E	0.631	1.061 (0.838–1.343)	0.254	0.852 (0.654–1.110)	1.25 × 10^−3^	1.946 (1.293–2.926)
	K/K vs. E/E	0.366	1.191 (0.840–1.688)	0.299	0.782 (0.512–1.193)	7.01 × 10^−5^	2.927 (1.748–4.901)
Dominant	E/K + K/K vs. E/E	0.465	1.089 (0.874–1.359)	0.164	0.837 (0.653–1.073)	6.07 × 10^−5^	2.161 (1.470–3.176)
Recessive	K/K vs. E/E + E/K	0.393	1.157 (0.832–1.611)	0.424	0.840 (0.559–1.262)	2.99 × 10^−3^	2.25 (0.291–3.175)

CI, confidence interval; DR, diabetic retinopathy; DWR, diabetes without retinopathy; NPDR, non-proliferative diabetic retinopathy; OR, odds ratio; PDR, proliferative diabetic retinopathy.

1 Study subjects comprised 933 DWR cases and 482 DR cases, of whom 140 presented with PDR, and 342 were diagnosed as NPDR.

2 Bivariate comparisons of allele and genotype frequencies were performed across retinopathy severity groups using Pearson’s *χ*^2^ test. Odds ratios (ORs) and 95% confidence intervals (CIs) are reported for each genetic model relative to the designated reference category.

### 3.4 Association between E23K genotypes and DR based on HbA1c levels

Stratified logistic regression analyses were performed by glycemic control (HbA1c ≤ 7.0% vs. >7.0%) to examine the association between E23K genotypes and DR (Table 4). In patients with HbA1c 7.0 or lower, using the E/E genotype as the reference, carriers of the E/K genotype had a significantly lower risk of DR (OR [95% CI] = 0.52 [0.37–0.73]), while individuals with the K/K genotype showed a notably higher risk (OR [95% CI] = 2.68 [1.45–4.95]). Conversely, in patients with HbA1c above 7.0, the E/K genotype was not significantly linked to DR (OR [95% CI] = 0.92 [0.66–1.28]). Still, the K/K genotype remained significantly associated with increased DR risk (OR [95% CI] = 2.51 [1.50–4.21]). After Benjamini–Hochberg FDR correction, associations with the K/K genotype remained significant in both HbA1c strata, whereas some initially significant E/K associations lost significance (Table 4). This supports a robust link between K/K homozygosity and greater retinopathy severity; other subgroup results should be viewed as exploratory, as HbA1c reflects not only glycemic status but also treatment, adherence, and lifestyle influences that were not fully accounted for in this dataset.

**Table 4:** Logistic regression analysis of the association between *KCNJ11* E23K genotypes and diabetic retinopathy stratified by glycemic control (HbA1c).^1^

Model		HbA1c ≤ 7.0^1^					HbA1c > 7.0^1^				
		DR^2^	DWR^2^	*p* ^1^	OR (95% CI)	FDR *p*^3^	DR^2^	DWR^2^	*p*	FDR *p*^3^	OR (95% CI)
Allelic	E	331 (67.8)^4^	550 (68.8)		1.00 (Reference)		315 (66.2)	765 (72.0)			1.00 (Reference)
	K	157 (32.2)	256 (31.8)	0.887	1.019 (0.801–1.297)	0.887	161 (33.8)	297 (28.0)	0.020	0.033	1.317 (1.044–1.661)
Genotypic	E/E	123 (50.4)	165 (41.0)		1.00 (Reference)		113 (47.5)	268 (50.5)			1.00 (Reference)
	E/K	85 (34.7)	220 (54.7)	0.004	0.52 (0.36–0.75)	0.008	89 (37.3)	229 (41.5)	0.620	0.689	0.92 (0.66–1.28)
	K/K	36 (14.9)	18 (4.3)	0.002	2.69 (1.45–4.98)	0.004	36 (15.3)	34 (8.0)	6.0 × 10^−4^	0.003	2.50 (1.49–4.19)
Dominant	E/E	123 (50.4)	165 (41.0)		1.00 (Reference)		113 (47.5)	268 (50.5)			1.00 (Reference)
	E/K + K/K	121 (49.6)	238 (59.0)	0.024	0.69 (0.50–0.95)	0.034	125 (52.5)	263 (49.5)	0.310	0.388	1.17 (0.87–1.57)
Recessive	K/K	36 (14.9)	18 (4.3)		1.00 (Reference)		36 (15.3)	34 (8.0)			1.00 (Reference)
	E/E + E/K	208 (85.1)	385 (95.7)	<0.001	3.70 (2.00–6.80)	0.003	202 (84.7)	497 (92.0)	2.0 × 10^−4^	0.002	2.59 (1.57–4.29)

CI, confidence interval; DR, diabetic retinopathy; DWR, diabetes without retinopathy; OR, odds ratio.

1 Logistic regression analyses stratified according to glycemic control status using HbA1c thresholds (≤7.0 and >7.0%). Odds ratios (ORs) and 95% confidence intervals (CIs) were calculated relative to the E/E reference genotype.

2 Nominal *p*-values were calculated using Fisher’s exact test for comparisons of genotype distributions within HbA1c strata. Odds ratios (ORs) and 95% confidence intervals (CIs) were estimated using logistic regression models with E/E as the reference genotype.

3 Benjamini–Hochberg false discovery rate correction was applied across all secondary HbA1c-stratified analyses.

4 Number (percent total).

### 3.5 Regression analysis

**Figure 1:**
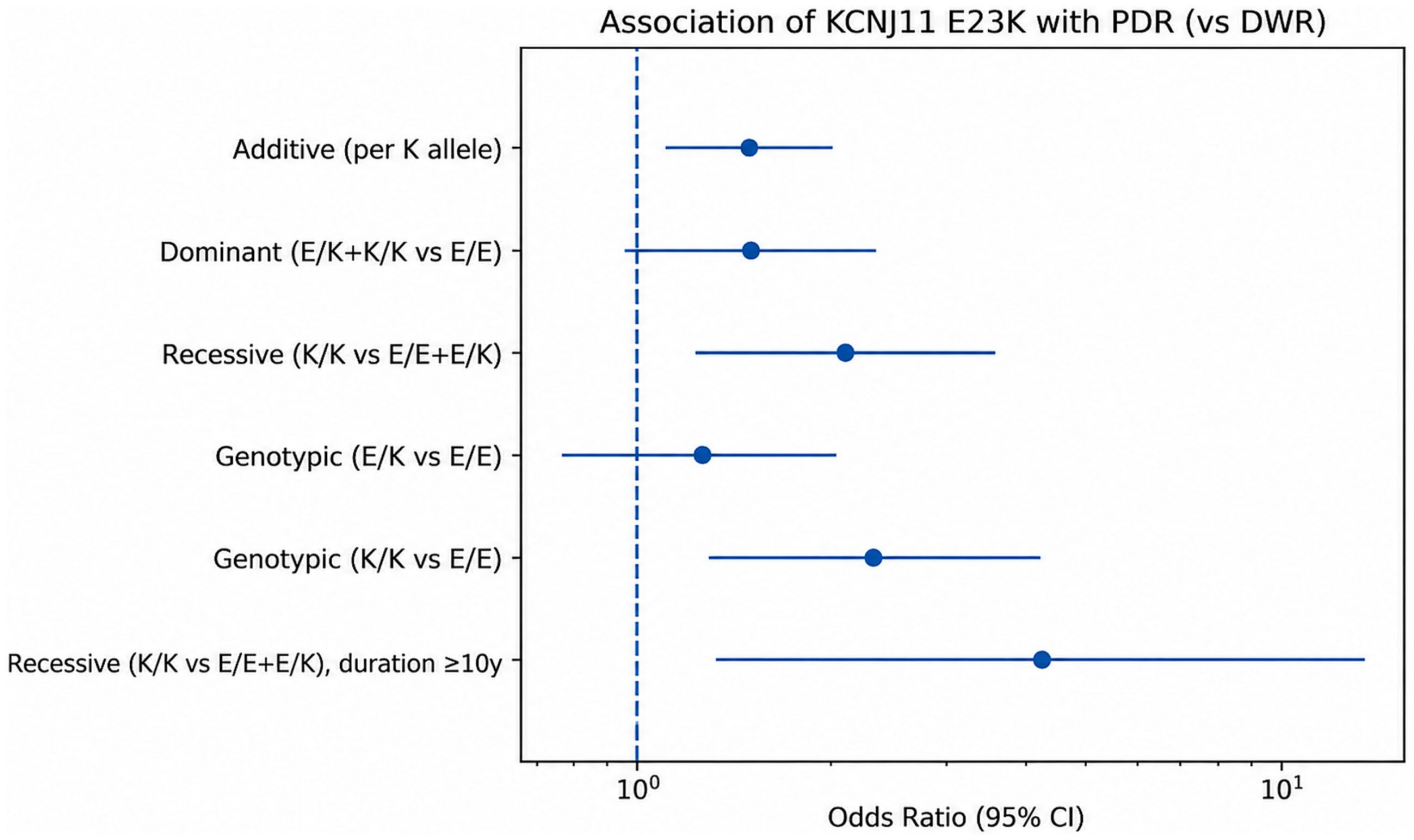
Forest plot showing the association between E23K and PDR compared with DWR across multiple genetic models. Odds ratios and 95% confidence intervals are presented for additive, dominant, recessive, and genotypic comparisons, with an additional recessive analysis stratified by diabetes duration (≥10 years). The vertical reference line indicates no effect (OR = 1).

Age- and sex-adjusted logistic regression showed that the E23K variant was linked specifically to the proliferative stage of retinopathy when compared with the DWR group (Table 5). Benjamini–Hochberg FDR correction was applied to all duration-stratified regression models. Associations that remained significant after adjustment are indicated in Table 5. In the additive model, each additional K allele increased the odds of PDR (OR [95% CI] = 1.49 [1.10–2.02]), consistent with a dose-dependent effect. The forest plot indicates that this pattern was driven largely by K/K homozygotes, as the estimates for the recessive and K/K genotype models were clearly shifted to the right of unity (Figure 1). Under the recessive model, K/K carriers had twice the odds of PDR (OR [95% CI] = 2.09 [1.22–3.58]; *p* = 0.008), and genotypic analysis confirmed risk was limited to K/K genotype carriers (OR [95% CI] = 2.34 [1.28–4.27]; *p* = 0.006), while heterozygotes were not at higher risk. The dominant model was not statistically significant. Although the inclusion of E23K genotype modestly increased the area under the ROC curve (ΔAUC = 0.046), the overall model performance remained poor (AUC range: 0.55–0.60), indicating limited discriminatory capacity. As such, these findings should be interpreted as evidence of a statistically detectable, but clinically modest, contribution of E23K to prevalent PDR classification.

**Table 5:** Multivariable logistic regression models evaluating the association between *KCNJ11* E23K and prevalent proliferative diabetic retinopathy.^1^

Model	OR	CI_low	CI_high	*p*	FDR *p*^2^	*N*
Allelic/genotypic model						
Additive (per K allele)	1.491	1.100	2.020	0.010	0.023	765
Dominant (E/K + K/K vs. E/E)	1.504	0.955	2.367	0.078	0.109	765
Recessive (K/K vs. E/E + E/K)	2.085	1.215	3.579	0.008	0.023	765
Genotypic (E/K vs. E/E)	1.255	0.765	2.059	0.368	0.429	765
Genotypic (K/K vs. E/E)	2.340	1.284	4.265	0.006	0.023	765
Duration-stratified recessive model (TT vs. CC + CT)						
Duration <10 years	1.036	0.286	3.759	0.957	0.957	102/32
Duration ≥10 years	4.227	1.311	13.625	0.016	0.028	101/38

CI, confidence interval; DWR, diabetes without retinopathy; OR, odds ratio; PDR, proliferative diabetic retinopathy.

1 Multivariable logistic regression analyses adjusted for age and sex. Additional subgroup analyses stratified by diabetes duration (<10 years vs. ≥10 years) were performed to evaluate potential duration-related differences in the association between *KCNJ11* E23K and prevalent PDR.

2 Benjamini–Hochberg FDR correction applied to all secondary regression analyses.

**Figure 2:**
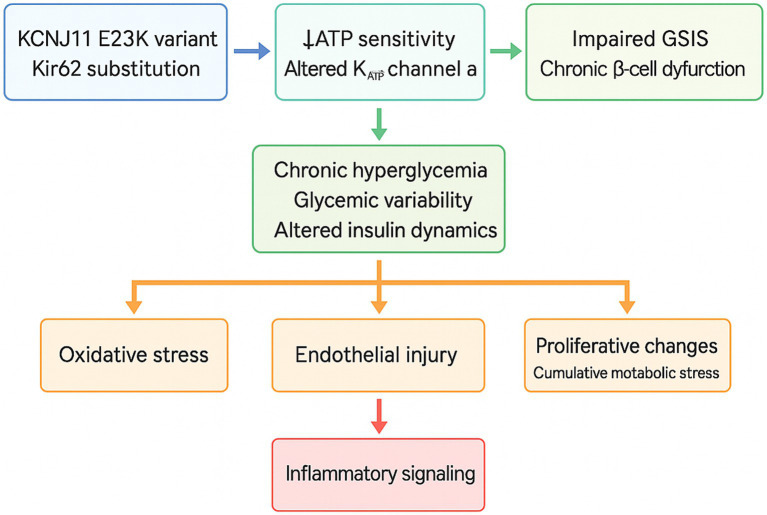
*KCNJ11* E23K variant drives a cascade from channel dysfunction to inflammation. *KCNJ11* E23K reduces ATP sensitivity of Kir6.2, impairing KATP channel closure and weakening glucose- stimulated insulin secretion. The resulting chronic β-cell dysfunction drives systemic metabolic stress: hyperglycemia, glycemic variability, and oxidative injury, that activates endothelial damage and inflammatory remodeling, ultimately promoting the neovascular changes characteristic of proliferative DR. Color coding denotes genetic (blue), cellular (green), metabolic (orange), and inflammatory (red) stages in this cascade.

Stratification by diabetes duration (<10 years vs. ≥10 years), implemented within regression models, further highlights effect modification. No association was observed when the T2DM duration was less than 10 years, while a significantly stronger recessive effect was evident in patients with a duration of ≥10 years (OR [95% CI] = 4.23 [1.31–13.63]; *p* = 0.016), as shown by the rightward shift in the estimate for duration ≥10 years (Figure 1). The recessive association observed among individuals with diabetes duration ≥10 years remained significant following FDR adjustment. Exploratory model-derived estimates suggested minimal differences in the predicted probability of prevalent PDR between genotype groups at shorter diabetes durations, whereas greater separation between genotype categories was observed at longer durations (Supplementary Figure S1). Because these estimates were generated from cross-sectional data and are dependent on model assumptions, they should not be interpreted as reflecting longitudinal trajectories or disease progression (Figure 2).

## 4 Discussion

This study provides the first evidence in a Middle Eastern population of a stage-specific association between the *KCNJ11* E23K variant and PDR, but with no evidence of association with earlier stages of retinopathy. The increased frequency of the K allele and the E/K and K/K genotypes in T2DM patients confirms that E23K is a susceptibility variant for diabetes and supports evidence that this mutation changes the Kir6.2 subunit (14, 18), reducing ATP sensitivity, impairing glucose-stimulated insulin secretion, and contributing to metabolic issues underlying T2DM and its complications (14, 31). Although no overall association was found between E23K allele frequencies and DR, genotype-based and stratified analyses revealed associations with proliferative and advanced stages, especially under poor glycemic control, indicating a potential gene–environment interaction associated with prevalent PDR rather than with the presence of overall retinopathy. Given the subtype-specific expression of *KCNJ11* in retinal tissue (32), its influence on proliferative disease is likely mediated indirectly through systemic metabolic stress, particularly glycemic variability, oxidative injury, and chronic inflammation, which, in turn, accelerate retinal neovascularization (28).

Consistent with earlier reports, higher frequencies of the K allele and the E/K and K/K genotypes were seen in individuals with T2DM than in control subjects (4, 11, 33–35), further establishing the role of *KCNJ11* E23K as a T2DM at-risk variant that modulates K_ATP_ channel activity, β-cell function, and release of insulin (36–38). On the other hand, comparable allele and genotype distributions were noted between the DR and DWR groups, suggesting that E23K is not an independent driver of early retinal microvascular changes. It is noteworthy that studies demonstrating positive associations likely reflect population-specific effects, limited statistical power, or context-dependent influences in which E23K contributes to retinopathy only under specific genetic or metabolic conditions (19, 39, 40).

Although not associated with overall DR, the E23K genotype was linked to DR severity, as evidenced by a significantly higher prevalence of the K/K genotype among patients with PDR. Few studies have explored the association between E23K and DR severity, and those that did were often underpowered or lacked stratification by metabolic control, potentially obscuring stage-specific genetic effects (41, 42). The consistent E23K allele frequencies across populations (19, 20, 24), combined with its variable associations with DR, imply that E23K is more of a stage-specific association with PDR than a primary factor in DR development. Although not tested here, this suggests that E23K may be preferentially associated with severe retinal disease under specific genetic or metabolic conditions, particularly in genetically predisposed individuals subjected to cumulative metabolic stress, rather than directly causing DR (22, 34, 36). As the severity analyses presented in Table 3 were based on unadjusted comparisons, the observed associations should be interpreted with caution until confirmed in multivariable models that account for established retinopathy risk factors.

Stratification by retinopathy severity revealed a clear link between the E23K variant and PDR, with the K allele and risk genotypes present only in PDR and absent in NPDR, indicating that *KCNJ11* E23K is associated with advanced vision-threatening disease rather than early retinal damage (25, 28). This highlights the importance of a variant associated with neovascularization and late-stage microvascular remodeling (19, 43). Glycemic-control stratification further demonstrated a nonlinear gene–environment interaction: K/K homozygosity increased risk regardless of HbA1c levels, whereas E/K was protective only when HbA1c was ≤7.0%, consistent with patterns observed at other DR-related loci (42, 43). HbA1c reflects clinical and behavioral influences such as medication use, diet, and physical activity, suggesting this finding reflects underlying heterogeneity rather than a direct causal effect. After Benjamini–Hochberg correction, only the most robust subgroup associations remained significant, supporting the main results while indicating that weaker findings are exploratory and should be considered exploratory and hypothesis-generating in the absence of detailed data on these factors. From a clinical perspective, genetic risk assessment could help identify well-controlled patients, especially K/K homozygotes, whose higher risk is not always reflected in standard measures.

Mechanistically, the E23K variant is associated with decreased ATP sensitivity of the Kir6.2 subunit, leading to altered K_ATP_ gating and, consequently, impaired glucose-stimulated insulin secretion (17, 18). The ensuing long-term β-cell dysfunction and fluctuations in blood glucose raise systemic metabolic stress (18), promoting oxidative stress, endothelial damage, and low-grade inflammation, key factors in retinal microvascular damage (6, 16). The heterogeneous expression of *KCNJ11* subtypes in the retina (32) suggests that these systemic effects likely worsen retinal pathology indirectly, particularly during neovascular stages (18, 44). As illustrated in Supplementary Figure S1, defective β-cell ATP sensing may intensify cumulative metabolic and vascular stress, thereby potentially contributing to biological processes associated with advanced, proliferative disease (17). It should be noted that these probability curves come from cross-sectional regression models and depend on their assumptions; thus, they are exploratory and do not demonstrate longitudinal patterns or causality, which require confirmation in longitudinal studies. Nonetheless, the model aligns with our findings, indicating a stronger effect of the E23K variant on proliferative than on non-proliferative disease.

Glycemic control is shaped by multiple intersecting factors, including pharmacotherapy, diet, physical activity, and adherence, each of which can influence metabolic status, retinopathy severity, and interactions with genetic variants such as *KCNJ11*. A principal limitation of this cohort is the absence of systematic medication data, which is particularly significant given that sulfonylureas act directly on K_ATP_ channels encoded by *KCNJ11* and may modify both glycemic levels and the functional expression of the E23K variant (45). By contrast, insulin and metformin likely affect retinal outcomes indirectly through improved glycemic control and vascular health (44), while antidiabetic therapies more broadly may influence glycemic and lipid profiles and alter progression to PDR (46). The gene–treatment interactions could not be formally evaluated in the absence of documented treatment data, and residual confounding from unmeasured medication effects cannot be excluded. Consequently, the HbA1c-stratified findings should be regarded as exploratory, and no causal inferences can be drawn regarding interactions among the E23K genotype, glycemic control, and prevalent proliferative diabetic retinopathy.

The observed association between the KCNJ11 E23K variant and prevalent severe DR underscores the importance of incorporating genetic markers, in addition to traditional metabolic factors, for more precise risk stratification. While the E23K genotype was associated with prevalent PDR in this cohort, its incremental contribution to model discrimination was modest. The observed increase in predictive performance (ΔAUC = 0.046) occurred within models demonstrating limited overall discrimination (AUC 0.55–0.60), underscoring that E23K genotyping alone is insufficient for clinical decision-making or targeted screening. Instead, E23K likely reflects a single element within a complex, multifactorial risk architecture that should be evaluated alongside established clinical, metabolic, and imaging markers (18, 25). Integration of the E23K status with clinical and metabolic risk factors is likely to improve early detection of individuals at higher risk of prevalent proliferative disease, thereby guiding personalized monitoring strategies (36).

This study’s strengths include a well-characterized, large Lebanese cohort with standardized ETDRS-based staging, enabling robust severity-specific analyses. By evaluating E23K in relation to both DR severity and glycemic control, we highlight clinically relevant gene-metabolic interactions that inform the relationship between genetic susceptibility and DR severity. The focus on an underrepresented Middle Eastern population addresses a major gap in the genetics of diabetic complications, while high-quality genotyping, stringent quality control, comprehensive phenotyping, and adjustment for key confounders strengthen the biological plausibility and translational relevance of the findings.

Several limitations must be recognized. A significant limitation is the absence of systematically recorded antidiabetic medication data. This is particularly relevant for sulfonylureas, which directly target KATP channels encoded by KCNJ11 and may modify the biological effects of the E23K variant, while insulin and metformin can influence retinopathy severity indirectly through improved metabolic control and vascular function. Because medication exposure was not documented, adjustment for therapy-related confounding was not possible, and separation of genetic from pharmacologic effects remains limited. The HbA1c-stratified findings should therefore be interpreted as exploratory, as observed genotype-specific associations may reflect unmeasured treatment effects rather than direct biological interactions, and the cross-sectional design precludes causal interpretation.

The cross-sectional design restricts causal inference and the assessment of progression from NPDR to PDR. Although E23K was associated with PDR, the lack of an association with NPDR and the absence of longitudinal data limit interpretation to stage-specific effects, highlighting the need for longitudinal studies. Despite the adequate overall sample size, subgroup analyses, especially for PDR, may have lacked the necessary power to detect modest genetic effects, gene–gene interactions, or clinical modifiers. Furthermore, functional validation was not performed, thus limiting mechanistic insights to established β-cell biology and indirect systemic pathways. Given the lack of an NPDR association and the cross-sectional design, progression from early to advanced retinopathy cannot be inferred; the PDR association is therefore stage-specific rather than longitudinal. Finally, replication across independent, ethnically diverse cohorts is essential, as population-specific allele frequencies, LD patterns, and gene–environment interactions may limit relevance beyond Middle Eastern populations.

## 5 Conclusion

The study indicates that the *KCNJ11* E23K variant is not associated with overall DR but is associated with prevalent PDR in a stage-specific manner. Given the cross-sectional design and the absence of association with earlier stages, no conclusions can be drawn regarding progression. These findings thus likely reflect underlying clinical heterogeneity and potential residual confounding from unmeasured medication exposures, particularly sulfonylurea use, and therefore require confirmation in prospective studies incorporating detailed treatment and lifestyle data. Patients with the K/K genotype exhibited a higher prevalence of advanced, vision-threatening DR, even when HbA1c levels are well managed. We propose that E23K represents one factor associated with prevalent PDR; however, its limited contribution to discrimination suggests that it should not be considered a standalone predictive marker. Future studies incorporating multi-marker approaches are needed to determine whether E23K provides clinically meaningful improvements in risk stratification.

## Data Availability

The datasets presented in this study can be found in online repositories. The names of the repository/repositories and accession number(s) can be found at: https://www.try-db.org/TryWeb/Home.php.

## Supplementary material

SUPPLEMENTARY FIGURE S1 Predicted probability of PDR across diabetes duration, stratified by *KCNJ11* E23K genotypes (E/E, E/K, K/K). Estimates are derived from cross-sectional logistic regression models. These exploratory curves rely on model assumptions and should not be interpreted as longitudinal trends, progression rates, or evidence of causality between disease duration and PDR risk.
