# Supplementary figures and images for: Association of the KCNJ11 E23K (rs5219) variant with proliferative diabetic retinopathy in Lebanese patients with type 2 diabetes
Source: Front Med (Lausanne). 2026 Jul 8;13:1874270. doi: 10.3389/fmed.2026.1874270 (PMC13388444; doi:10.3389/fmed.2026.1874270)

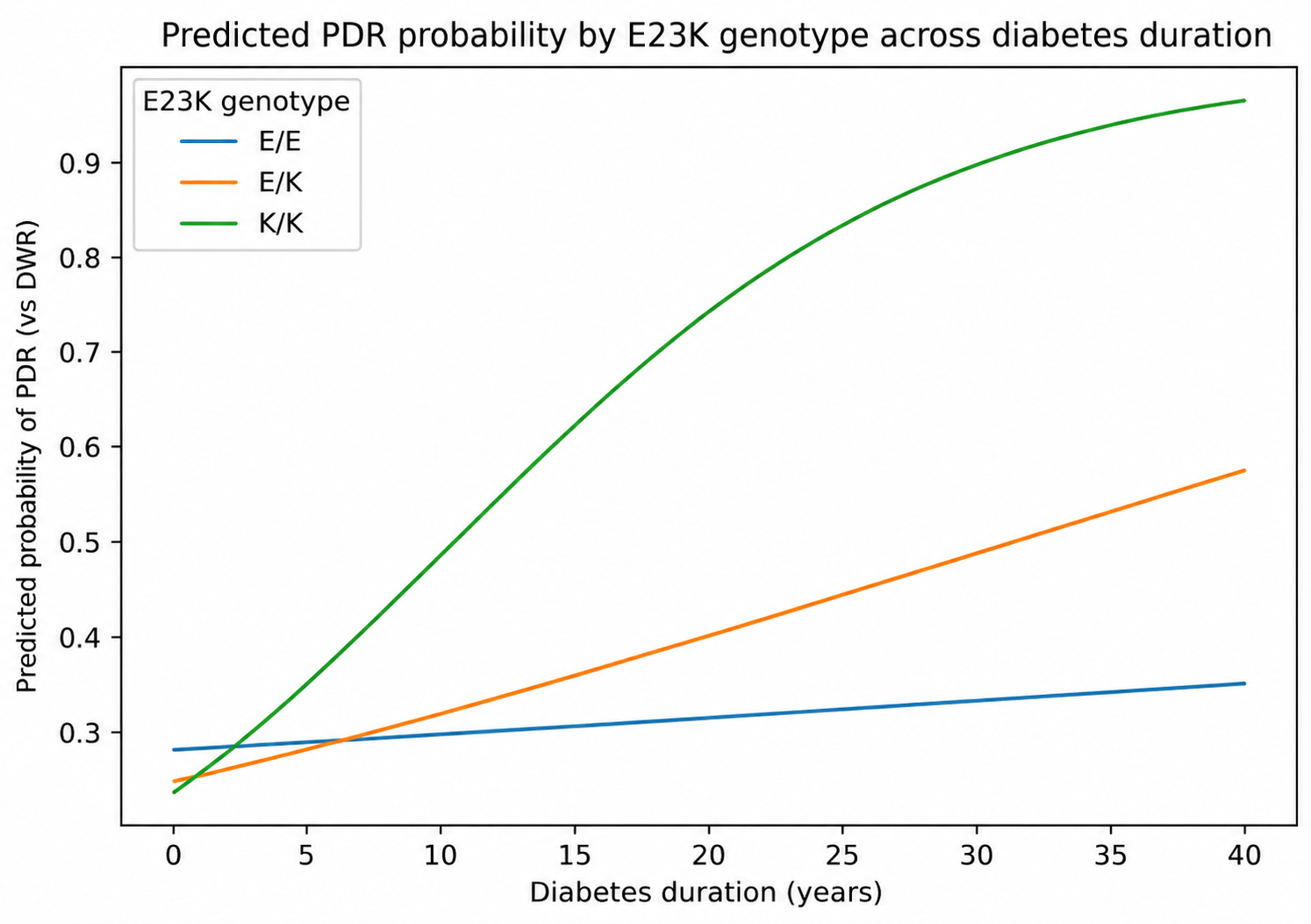

Supplement: SUPPLEMENTARY FIGURE S1 — Predicted probability of PDR across diabetes duration, stratified by KCNJ11 E23K genotypes (E/E, E/K, K/K). Estimates are derived from cross-sectional logistic regression models. These exploratory curves rely on model assumptions and should not be interpreted as longitudinal trends, progression rates, or evidence of causality between disease duration and PDR risk. [file Image_1.tiff]
